# Generation of mutant mice via the CRISPR/Cas9 system using FokI-dCas9

**DOI:** 10.1038/srep11221

**Published:** 2015-06-09

**Authors:** Satoshi Hara, Moe Tamano, Satoshi Yamashita, Tomoko Kato, Takeshi Saito, Tetsushi Sakuma, Takashi Yamamoto, Masafumi Inui, Shuji Takada

**Affiliations:** 1 Department of Systems BioMedicine, National Research Institute for Child Health and Development, Tokyo 157-8535, Japan; 2 Department of Mathematical and Life Sciences, Graduate School of Science, Hiroshima University, Hiroshima 739-8526, Japan

## Abstract

Genome editing, which introduces mutations in genes of interest using artificial DNA nucleases such as the ZFN, TALEN, and CRISPR/Cas9 systems in living cells, is a useful tool for generating mutant animals. Although CRISPR/Cas9 provides advantages over the two other systems, such as an easier vector construction and high efficiency of genome editing, it raises concerns of off-target effects when single guide RNA (gRNA) is used. Recently, FokI-dCas9 (fCas9), a fusion protein comprised of the inactivated mutant form of Cas9 and the DNA nuclease domain of FokI, has been developed. It enables genome editing with reduced risks of off-target effects in mammalian cultured cell lines, as fCas9 requires gRNAs to bind opposite strands with an appropriate distance between them. Here, we demonstrated that fCas9 efficiently generates living mutant mice through microinjection of its mRNA and gRNAs into zygotes. A comparison of the relative efficiencies of genome editing using fCas9 and other modified Cas9s showed that these mutagenesis efficiencies are similar when the targets of two gRNAs are separated by an appropriate distance, suggesting that in addition to the ease of vector construction, fCas9 exhibit high efficiency in producing mutant mice and in reducing risks of off-target effects.

Loss-of-function studies are essential approaches to understand the role of a gene of interest *in vivo*. Thus far, gene targeting based on homologous recombination (HR) in embryonic stem (ES) cells has been used to generate knockout mice carrying mutations in a gene of interest^1^. Although it represents one of the most important technologies, it is a time-consuming and labor-intensive process to produce mutant ES cells, and chimeric mice, in the germline of which these mutant ES cells are inserted. Recently, a new technology to produce knockout mice, which minimizes the cost as well as the handling procedure and time, has been developed based on genome editing. Mutations are induced through the introduction of artificial DNA nucleases, such as zinc-finger nuclease (ZFN), transcription activator-like effector nuclease (TALEN), and clustered regulatory interspaced short palindromic repeat (CRISPR)/CRISPR-associated (Cas), into zygotes and cultured cells, including ES cells. ZFN and TALEN are fusion proteins comprising sequence-specific DNA-binding domains (recognizing 12–20 nt and 16–20 nt, respectively) and the dimeric DNA nuclease domain of FokI. When two ZFNs or TALENs bind specific loci separated by an appropriate distance in the genome, a double-strand break (DSB) is caused by the dimerized FokI, which induces either HR or non-homologous end-joining (NHEJ), of which the latter represents an error-prone DNA repair pathway that generates insertion or deletion (indel) mutations, resulting in a frameshift mutation in the gene^2^,^3^. In addition, ZFNs or TALENs can induce HR-mediated substitutions in a gene in the nucleus of a living cell, when introduced together with an oligo DNA or a double-stranded DNA as a donor^3^,^4^. An advantage of using TALEN over ZFN is that TALEN plasmids can be easily constructed in a molecular biology laboratory. Especially, Golden Gate TALEN system (Golden TALEN) enables us to construct TALEN plasmids easily^5^,^6^.

The CRISPR/Cas9 system consists of RNA-guided Cas9 from the *Streptococcus pyogenes* CRISPR system, and chimeric guide RNA (gRNA), which includes the 20-nt sequence of a target genomic locus, with an NGG protospacer-adjacent motif (PAM)^7^,^8^. Cas9 and gRNA together form a complex that hybridizes to the target genomic sequence, and Cas9 subsequently introduces the DSB. The resulting cleft is repaired by NHEJ or HR, so that CRISPR/Cas9 can induce a mutation in the genomic locus of interest, as is the case with ZFN and TALEN. Previously we have utilized two of above genome editing techniques, Golden TALEN and CRISPR/Cas9, to produce mutant mice and found two advantages of the CRISPR/Cas9 system over that TALEN; the vector construction of the CRISPR/Cas9 system is easier than that TALEN, and the efficiency of genome editing using CRISPR/Cas9 was higher than that of TALEN in our experimental set-up^9^,^10^,^11^. Recently, Sakuma *et al.* introduced a modified form of the TALEN system called Platinum TALEN, with high efficiency of genome editing in *Xenopus* and in rats^12^. Also in mice, several studies have been reported the production of mutant animals using Platinum TALEN^13^,^14^. However, there have been no reports which directly compared the effectiveness of the Golden and Platinum TALEN systems in producing mutant mice via microinjection into fertilized eggs.

Genome editing enables the production of ES cell-independent knockout animals via the microinjection of the mRNAs from these artificial DNA nucleases into fertilized eggs^15^,^16^,^17^. This approach has several advantages over traditional ES cell-mediated methods, such as easier construction of plasmid vectors for the TALEN and CRISPR/Cas9 systems, no requirement of a chimeric mouse, and faster and cheaper production of knockout mice. In addition, genome editing enables the disruption of a gene that has a unique sequence, but is surrounded by repeat units such as Y-linked genes, as the artificial DNA nuclease used for genome editing requires less than 60 nt of specific sequence^18^. In such cases, it is difficult to generate a knockout mouse using traditional ES cell-mediated methods, because it requires several kb of homologous arms. Indeed, we previously showed that the Y-linked *Sry* gene could be disrupted via microinjection of TALENs into fertilized eggs^10^.

Although genome editing technologies facilitate the production of knockout mice, there is increased concern regarding off-target effects when the CRISPR/Cas9 system is used, as gRNAs recognize ≈20 nt while allowing some mismatches^19^. On the other hand, the ZFN and TALEN systems should theoretically have low levels of off-target effects, as the two units bind to opposite strands at an appropriate distance from each other to induce a DSB. To overcome the off-target effects of the CRISPR/Cas9 system, two modified forms of Cas9 have been developed, namely, Cas9 D10A and FokI-dCas9 (fCas9)^20^,^21^,^22^. Cas9 D10A cannot induce DSBs, but contains the nickase activity. DSBs can only be induced when Cas9 D10A is introduced into the nucleus with two gRNAs, with both of them binding in the vicinity of a genomic locus on opposite strands^20^. fCas9 is a fusion protein comprising an inactivated mutant form of Cas9 (dCas9) and the FokI DNA nuclease. DSBs can only be induced when fCas9 is introduced into the nucleus with two gRNAs; the latter bind at an appropriate distance from each other on a genomic locus on opposite strands^21^. Theoretically, genome editing using fCas9 has lower risks of off-target effects than that using Cas9 D10A, as the range of distances between the two binding gRNAs that is required for DSBs is narrower in fCas9 (14–17 bp) than in Cas9 D10A (4–20 bp)^20^,^21^,^22^. The adoption of fCas9 and gRNAs could be a good choice for the generation of knockout mice from fertilized eggs, with ease of vector construction and reduced risks of off-target effects. As genome editing utilizing fCas9 and gRNAs has only been tested in cultured cell lines, we examined whether this system could be applied to the production of knockout mice via microinjection into fertilized eggs. In addition, we compared the efficiency of genome editing using the fCas9 system with that of various TALEN systems.

## Results

**Figure 1:**
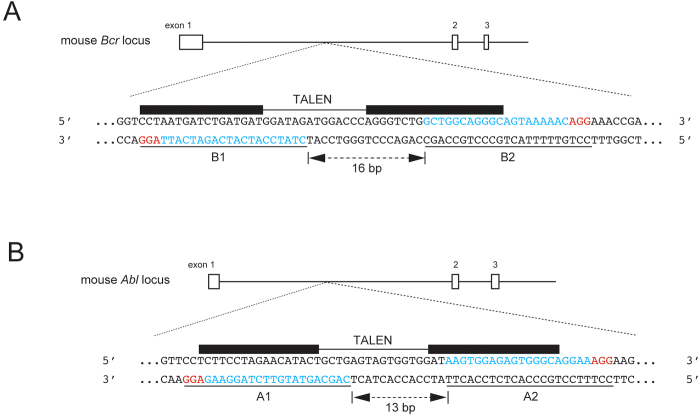
Schematic representation of the Bcr and Abl loci. (**A**) Designs of the gRNAs and TALENs on the mouse *Bcr* locus. A part of the genomic structure of the *Bcr* locus is shown at the top. The white boxes and lines represent exons and introns, respectively. The nucleotide sequence of the target of genome editing is indicated at the bottom. Black boxes depict the target sequences of the TALENs. Blue and red underlined letters indicate the target sequences of gRNAs and PAMs, respectively. The position and length of spacers for the gRNAs are shown with a double-headed arrow with letters. (**B**) Designs of gRNAs and TALENs on the mouse *Abl* locus. A part of the genomic structure of the *Abl* locus is shown at the top. White boxes and lines represent exons and introns, respectively. The nucleotide sequence of the target of genome editing is indicated at the bottom. Black boxes depict target sequences of the TALENs. Blue and red underlined letters indicate the target sequences of gRNAs and PAMs, respectively. The position and length of spacers for the gRNAs are shown with a double-headed arrow with letters.

**Figure 2:**
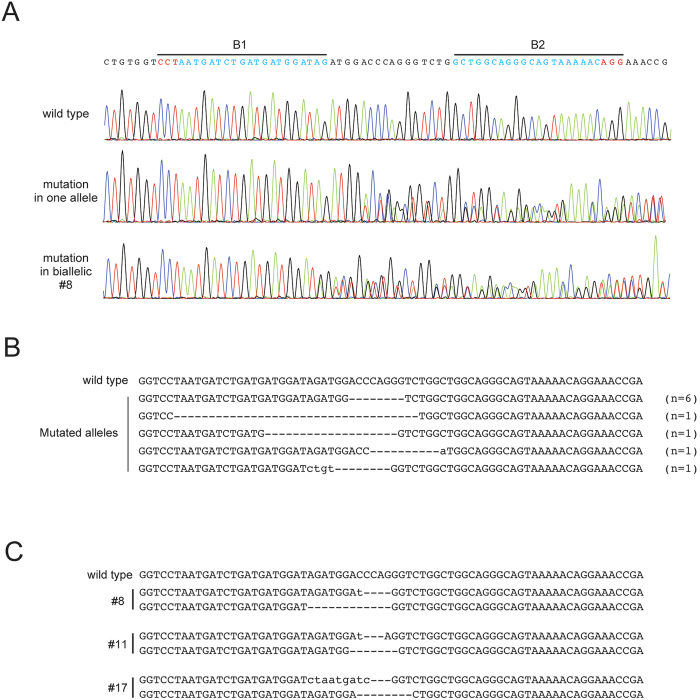
Allelic sequences of mice mutated on the Bcr locus obtained using the CRISPR/Cas9 system with fCas9. (**A**) Electropherograms around the target sequences of the gRNAs of wild-type and mutant mice. Wild-type sequences are indicated at the top with lines showing the positions of the target sequences of the gRNAs. (**B**) Allelic sequences of wild-type and heterozygous mutants identified in Trial 1. Hyphens and small-case letters indicate deleted and inserted sequences, respectively. The numbers of mutant alleles are indicated at the right of each mutant sequence. (**C**) Allelic sequences of biallelic mutants identified in trial 1. The sequences of both alleles in individual mice are shown with their identification numbers. Hyphens and small-case letters show deleted and inserted sequences, respectively. A wild-type sequence is indicated at the top as a reference.

**Figure 3:**
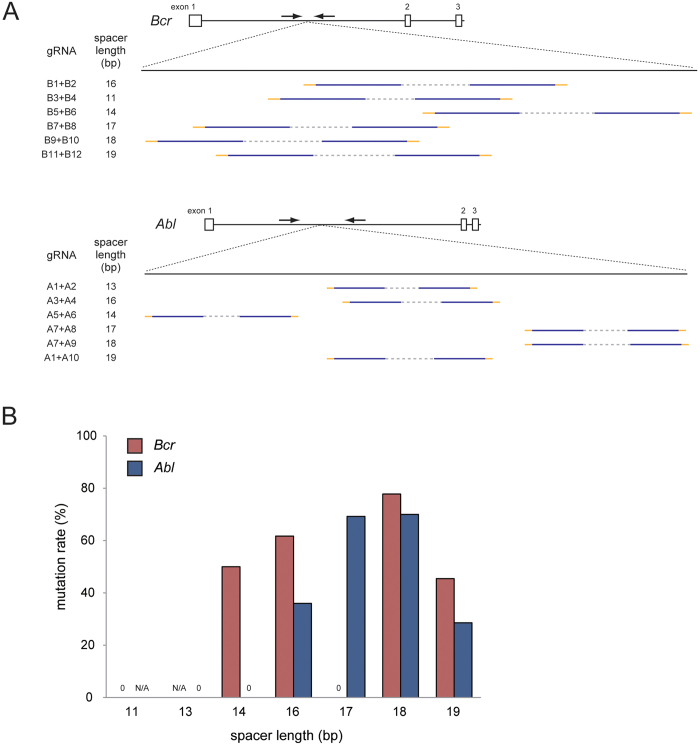
Comparison of the spacer length in the catalytic activities of fCas9. (**A**) gRNA designs for *Bcr* (top) and *Abl* (bottom) loci. The white boxes and lines represent exons and introns, respectively. Arrows indicate primers for genotyping analysis. Blue and orange lines indicate the target sequences of gRNAs and PAMs, respectively. Gray dashed lines indicate spacers of gRNA pairs. The lengths of spacers are shown in left. (**B**) Mutation efficiencies of embryos injected gRNA pairs with different spacer length. Mutation rates of each gRNA pair are indicated as red (*Bcr*) and blue (*Abl*) bars, respectively. N/A; not applicable.

To assess whether mutant mice can be generated by microinjection of fCas9 and gRNAs into fertilized eggs, a pair of gRNAs (B1 and B2) were designed at opposite DNA strands with a 16-bp spacer length on an intronic region of the *Bcr* locus (Fig. 1A). After microinjection and the transfer of two-cell stage embryos into a pseudopregnant female, the genotypes of embryos or newborn pups were determined. We obtained 16, 12, and 19 embryos or newborns from 114, 32, and 67 microinjected eggs, respectively, from three trials. The survival rates of the embryos were 14.0%, 37.5%, and 28.4%, respectively (Table 1). Some of these survival rates are within a range similar to that obtained when a single gRNA and human codon-optimized, wild-type Cas9 (Cas9 WT) was microinjected (a total of 15 and 16 embryos were obtained from B1/Cas9 WT microinjection into 46 eggs, and from B2/Cas9 WT microinjection into 40 eggs, and the respective survival rates were 32.6% and 40.0%; Table 1). The average of survival rates using fCas9 and all the Bcr gRNAs in this study showed slightly lower than that of Cas9 WT (fCas9 vs. Cas9 WT; 23.2 ± 10.4% vs. 33.0 ± 8.5% (mean ± SD); *P* = 0.009, χ^2^ test), suggesting that injection of fCas9 mRNA led to toxicity to some extent for mouse eggs and embryos at each of the concentrations that we used. Electropherograms obtained by PCR-direct sequencing of the genomic DNA obtained from embryos microinjected with fCas9 and gRNAs (B1 and B2) showed overlapping peaks around the gRNA target sequences (Fig. 2A) in 29 out ofthe 47 (61.7%) genotyped embryos (Table 1), including 26 of the monoallelic (Fig. 2A,B) and 3 of the biallelic (Fig. 2A,C) mutants. This was comparable to the rates obtained following B1/Cas9 WT and B2/Cas9 WT microinjection (66.7% and 62.5%, respectively; Table 1). This result shows that genome editing by using gRNAs/fCas9 exhibits a comparable efficiency in producing knockout mice with that of microinjection of RNAs into fertilized eggs by using a single gRNA/Cas9 WT.

In order to compare the efficiency of genome editing using fCas9 and Cas9 D10A, respectively, the same pair of gRNAs used for the above experiment (B1 and B2) and Cas9 D10A were microinjected into fertilized eggs, as in the gRNAs/fCas9 experiments. We obtained 15 embryos out of 54 (27.8%) microinjected eggs (Table 1); this is comparable to the result obtained from microinjecting gRNA/Cas9 WT (Table 1). Genotyping analysis showed that the rate at which we obtained mutants seemed to be lower in gRNAs/Cas9 D10A-microinjected embryos (5 mutants out of 15 embryos, or 33.3%, Table 1) than that in the three trials of gRNAs/fCas9 microinjection (Table 1). It might be possible that microinjection of fCas9 exhibits similar or better genome editing efficiency than that of Cas9 D10A.

Furthermore, to examine whether the distance separating the recognition sequences of a pair of gRNAs influences the genome editing activity of fCas9, we designed two gRNAs (A1 and A2) in the mouse *Abl* locus with 13 bp of spacer length, and repeated the same microinjection experiments as those for the *Bcr* region, using those two gRNAs (Fig. 1B). The genotyping analysis showed that all of the 52 embryos were wild-type, and no mutant was obtained (Table 2). This could be attributed to the inappropriate design of one or both of the gRNAs. This possibility was examined by using microinjection of A1/Cas9 WT, A2/Cas9 WT, and A1/A2/Cas9 D10A. Genotyping analyses of the embryos thus obtained showed that 80% of A1/Cas9, 46.2% of A2/Cas9, and 50% of A1/A2/Cas9 D10A-microinjected embryos were mutant (Table 2), suggesting that the lack of mutant production by A1/A2/fCas9 injection was not due to the gRNA sequence. It can be possible that the spacer length of the pair of gRNAs (13 bp) was inappropriate for the functioning of fCas9. To examine this possibility, we microinjected additional gRNA pairs for *Bcr* and *Abl* loci which have various spacer length from 11 to 19 bp (B3–B12 and A3–A10; Fig. 3A) with fCas9 mRNA. Genotyping analyses of embryos injected gRNAs for *Bcr* locus showed that no mutants were obtained in two gRNA pairs with 11 and 17 bp spacer length, while 50%, 78% and 45% of embryos injected gRNA pairs spaced 14, 18 and 19 bp were mutated, respectively. Same analyses in *Abl* locus showed that embryos injected with a gRNA pair with 14 bp of spacer length were not mutated, whereas 36%, 69%, 70% and 29% of embryos were mutated when spacer length of injected gRNA pairs were 16, 17, 18 and 19 bp, respectively (Fig. 3B and Supplementary Table S1). We also analyzed mutation efficiencies of embryos injected gRNA B3, B4, A3 and A4 with Cas9 WT as the above experiments. As a result, we confirmed the functions of gRNA B3, B4 and A3 to recruit Cas9 and induce indels to target loci (Supplementary Table S2). No embryos injected A4/Cas9 WT were obtained from pseudopregnant female. It could be possible that combination of A4/Cas9 WT is toxic for embryos since no embryo injected with A4/Cas9 WT was alive after three days of culture. Taken together, these results suggested that the DNA nuclease activities of fCas9 depend on the distance of spacer length between recognition sequences of a gRNA pair.

It has been reported that fCas9 has higher specificity compared with Cas9 WT and D10A in cultured cell lines^20^,^21^. To confirm if it is the case in embryos injected with gRNAs and Cas9, we analyzed off-target effects in embryos injected gRNA pair with 16 bp spacer length in *Bcr* and *Abl* loci (B1/B2 and A3/A4, respectively). Three possible off-target sites for B1 and B2, which were gRNA pairs used for mutants in *Bcr* locus with fCas9, were computationally searched. Sequencing analysis in a total of 282 loci (three possible off-target sites for each gRNA for 47 injected embryos) showed that any off-target mutation was not observed. We also analyzed in B1/B2/Cas9 D10A-, B1/Cas9 WT- and B2/Cas9 WT-injected embryos, and no off-target mutation was found in any cases (Supplementary Table S3). The same analyses were also performed in A3 and A4 on *Abl* locus. Total of 150 loci in A3/A4/fCas9- and A3/Cas9 WT-injected embryos (two or three possible off-target sites for A3 or A4 for 25 or 15 injected embryos, respectively) were analyzed, and no off-target mutation was observed (Supplementary Table S4). Taken together, these results indicated that the specificity of fCas9 is also high as well as other Cas9s in generation of mutant mice using microinjection into zygotes.

Finally, we compared the respective efficiencies of genome editing among the Platinum Gate TALEN, Golden Gate TALEN and CRISPR/Cas9 systems using fCas9. To this end, the Platinum and Golden Gate TALENs were assembled to recognize the same sequences in close vicinity to the gRNAs used for the above experiments (using the intronic sequences of *Bcr* and *Abl,* Fig. 1A, B). For a precise comparison of their activities, it is ideal to design TALENs and gRNAs to recognize exactly the same sequence; however, this is impossible as TALENs and gRNAs require a T and NGG, respectively, at their 3’ ends. The microinjection of Platinum and Golden Gate TALENs and the two-cell stage embryo transfer were performed, and the genotype of each embryo obtained was determined by PCR-direct sequencing. On the *Bcr* locus, the efficiencies of mutagenesis were 50.0% and 15.4% for the Platinum and Golden Gate TALENs, respectively (Supplementary Table S5). On the *Abl* locus, the efficiency of mutagenesis was 64.7% when the Platinum Gate TALEN was microinjected, whereas no mutant was obtained from the Golden Gate TALEN microinjection (Supplementary Table S5). The Platinum Gate TALEN showed higher efficiency of genome editing than the Golden Gate TALEN, and similar efficiency to that of the CRISPR/Cas9 system using fCas9.

## Discussion

It has been shown that fCas9 is useful for genome editing in human cultured cell lines with a >140-fold higher specificity than Cas9 WT, and its efficiency for genome editing is similar to that of Cas9 D10A^20^,^21^,^22^. This study demonstrates that fCas9 is appropriate for the generation of knockout mice using microinjection of mRNA-coding fCas9 and two gRNAs into fertilized eggs. In addition fCas9, Cas9 WT, and Cas9 D10A showed similar efficiencies of genome editing in our study. It might be possible that the nuclease domains of fCas9 and Cas9 WT have similar efficiencies in digesting the genome. It has been reported that fCas9 requires a spacer distance of 14–17 bp by EGFP disruption assay, whereas more than 19 bp of spacer can induce indels to endogenous genomic loci in human cultured cells^21^,^22^. Our results using mice zygote also observed that gRNA pairs with 14–19 bp of spacer length introduced mutation with fCas9, while no mutants were detected when the spacer length was less than 13 bp. These results indicate that the spacer length of gRNA pairs have to be designed more than 14 bp for producing mutant mice using fCas9.

It must be noted that mutations can exist outside of the target loci, called “off-target effects,” when mutant cells or animals are generated using a single gRNA and Cas9 WT, because the recognition sequence is less than 20 nt while allowing some mismatches. In fact, Fu *et al.* showed that they identified off-target effects in cultured cell lines^19^. It has been reported the risks of off-target effects of fCas9 is much lower than Cas9 WT and even Cas9 D10A^20^,^21^,^22^. On the other hand, recent studies reported that off-target effects are rare in mutant mice generated by microinjection using the CRISPR/Cas9 system into mouse zygotes^23^,^24^. Our analyses support this in addition fCas9 injected embryos has also low risks of off-target effects. Nevertheless, it is better to use a modified Cas9 system such as Cas9 D10A or fCas9 with reduced risks of off-target effects, and to postulate that there are unknown mutations in mutants generated using the CRISPR/Cas9 system, because whole-genome sequencing to identify a mutant without any off-target effect is considerably expensive. In this study, we showed that fCas9 can be applied to the production of knockout mice at similar efficiencies and is to those exhibited by Cas9 WT and Cas9 D10A, suggesting that fCas9 should be the fusion protein of choice to minimize off-target effects.

It has been reported that mutations can be induced by genome editing using the Golden Gate TALEN system with high efficiency in a variety of species, such as the zebrafish or *Xenopus*; however, its efficiency is not comparable in mammals^25^,^26^,^27^. Recently, the Platinum Gate TALEN system has been developed, which can efficiently induce mutations in rats following its microinjection into their zygotes^12^. This observation is also applicable to mouse models, as it is consistent with our results of producing knockout mice on the *Bcr* and *Abl* loci using the Golden and Platinum Gate TALEN systems. Our data also implies that Platinum Gate TALEN and CRISPR/Cas9 exhibit similar efficiencies of genome editing using fCas9. The use of both the systems offers flexibility in the selection of target sequences for the generation of knockout mice with reduced risks of off-target effects. gRNAs in the CRISPR/Cas9 system used with fCas9 require two NGG sequences (PAM) at opposite strands that are sufficiently apart within a narrow range, which is difficult to design within AT-rich genomic loci. The Platinum Gate TALENs can be designed with more flexibility than gRNAs for the CRISPR/Cas9 system using fCas9, because they require two Ts at opposite strands that are adequately apart within a narrow range. However, it is sometimes difficult to design target sequences on GC-rich regions such as CpG islands. Since genomic loci that are difficult to disrupt using the CRISPR/Cas9 system with fCas9 could be targeted using the Platinum Gate TALEN system and *vice versa*, selecting a suitable application is crucial for flexible genome editing.

In summary, we showed that the generation of mutant mice using the CRISPR/Cas9 system with fCas9 could be achieved via microinjection into zygotes. The specificity of fCas9 is more strictly regulated than that of other Cas9s, because the two gRNAs recognize genomic loci at opposite strands that are separated by an appropriate distance, within a small range. The CRISPR/Cas9 system with fCas9 enables us to generate knockout mice with reduced risks of off-target effects and with ease of vector construction.

## Materials and methods

### Plasmids

Plasmids containing fCas9, Cas9 WT, Cas9 D10A (human codon optimized) and gRNA cloning vectors were obtained from Addgene (Plasmid #52970, #41815, #41816, and #41824, respectively). The gRNA cloning vector was used with some modifications, as described by Inui *et al.*^11^ A Golden Gate TALEN and TAL Effector Kit was obtained from Addgene^5^. The Platinum Gate TALEN used is described by Sakuma *et al.*^12^

### gRNA target sequences

Following sequences were used for the target of gRNAs in this study: B1; 5’-CTATCCATCATCAGATCATTAGG-3’, B2; 5’-GCTGGCAGGGCAGTAAAAACAGG-3’, B3; 5’-CATCAGATCATTAGGACCACAGG-3’, B4; 5’-GACCCAGGGTCTGGCTGGCAGGG-3’, B5; 5’-GGACCACAGGCATGACAGAACGG-3’, B6; 5’-AGATGGACCCAGGGTCTGGCTGG-3’, B7; 5’-ACAGGCATGACAGAACGGGCTGG-3’, B8; 5’-GATGATGGATAGATGGACCCAGG-3’, B9; 5’-TGCCCTGCCAGCCAGACCCTGGG-3’, B10; 5’-AATCTTTTGAAAATAATCCAGGG-3’, B11; 5’-AGAACGGGCTGGCACGCTGCAGG-3’, B12; 5’-ATGATCTGATGATGGATAGATGG-3’, A1; 5’-CAGCAGTATGTTCTAGGAAGAGG-3’, A2; 5’-AAGTGGAGAGTGGGCAGGAAAGG-3’, A3; 5’-ACTACTCAGCAGTATGTTCTAGG-3’, A4; 5’-GTGGGCAGGAAAGGAAGAGGAGG-3’, A5; 5’-AGAGTGGGCAGGAAAGGAAGAGG-3’, A6; 5’-TCAAAAGTAAAGGGCTGGCTGGG-3’, A7; 5’-CCACAATGAGAACTGTTACTCGG-3’, A8; 5’-ATTTCCCAGAGGAAAGAAATGGG-3’, A9; 5’-AGGTGTGCCGACTCTCCTCATGG-3’ and A10; 5’-GGTGTGCCGACTCTCCTCATGGG-3’.

### Construction of gRNA and TALEN expression vectors

gRNA sequences were cloned by inverse PCR using a gRNA cloning vector and primers for gRNA cloning (Supplementary Table S6), followed by *Dpn*I (New England Biolabs Inc., Beverly, MA, USA) digestion and transformation into DH5α, as previously described^11^. The sequences of the gRNAs were verified by sequencing analysis. The primers used were listed on supplementary Table S6. The specific target site of TALEN was researched using the TALEN Targeter 2.0 software program (https://tale-nt.cac.cornell.edu/node/add/talen-old). Construction of the TALEN vectors were performed as previously described^12^.

### RNA synthesis

Two μg of plasmids coding for TALENs and fCas9, which contained the T7 RNA promoter sequence, were linearized with *Sph*I (Takara Bio) and *Age*I (Wako), respectively, and used as templates for RNA synthesis. The T7 RNA promoter sequence was added by PCR amplification of the gRNAs, Cas9 WT, and Cas9 D10A-coding plasmids. The primers used were listed on supplementary Table S6. The primer set T7-Cas9 was used for amplification of Cas9 WT and Cas9 D10A. The PCR products were used as templates for RNA synthesis. The RNAs were synthesized with the mMESSAGE/mMACHINE T7 Kit (Ambion, Austin, TX, USA), and purified using the MEGAclear RNA Purification Kit (Ambion) according to the manufacturer’s instructions.

### Microinjection

F1 female mice obtained from the mating of C57BL/6 × DBA2 (B6D2F1) mice were superovulated by an injection of 5 IU of pregnant mare’s serum gonadotropin and human chorionic gonadotropin according to standard procedures. Superovulated B6D2F1 females were crossed with B6D2F1 males, and fertilized eggs at the pronucleus stage were collected in M2 medium. For the single gRNA/Cas9 mRNA, RNA concentrations used for microinjection were 500 ng/μl in total (1:1 ratio, 250 ng/μl each). For gRNAs/Cas9 WT, gRNAs/Cas9 D10A, and gRNAs/fCas9, mRNAs were mixed at 1:1:1 ratio in a total concentration of 500 ng/μl (167 ng/μl each). For the Golden and Platinum Gate TALENs, mRNAs were mixed at 1:1 ratio at a total concentration of 1 μg/μl (500 ng/μl each) and 25 ng/μl (12.5 ng/μl each), respectively. RNAs were injected into the cytoplasm of zygotes using FemtoJet microinjector (Eppendorf, Westbury, NY, USA). The microinjected embryos were incubated in KSOM medium at 37 °C for one day and subsequently transferred into pseudopregnant ICR female mice at the two-cell stage. All mice were purchased from the Sankyo Labo Service Corporation (Tokyo, Japan). Animal protocols were approved by the Animal Care and Use Committee at the National Research Institute for Child Health and Development. All experiments were conducted in accordance with these approved animal protocols.

### Genotyping

Genomic DNA was extracted from the pre- and postnatal embryos. The genomic regions around the gRNAs were amplified by PCR using BIOLINE Taq (BioLine, London, UK) and the primers for the *Bcr* and *Abl* region, respectively (Supplementary Table S6). For direct sequencing, PCR products were treated with ExoSAP-IT (USB) and subjected to sequencing analysis. The genotypes were determined from electropherograms. When overlapping peaks were observed around the gRNA target sequences in an electropherogram, the genotype was considered to be mutant. In cases where there were no overlapping peaks and the read sequence was the same as that of a wild-type, the genotype was considered to be wild-type. Next, we examined whether wild-type sequences could be followed by overlapping peaks. When a wild-type sequence existed, it was considered to be a monoallelic mutant. In case a wild-type sequence could not be followed, it was considered to be a biallelic mutant. For the determination of the nucleotide sequence of each mutated allele, the PCR products were cloned into a pGEM-T Easy Vector (Promega Corp., Madison, WI, USA) and sequenced. Mosaic animals containing small contributions of mutated alleles, determined from the electropherograms, were counted as wild-type mice.

### Off-target analysis

Putative off-target sites of B1, B2, A3 and A4 were searched using online-based web tool (http://genome-engineering.org/) and picked up two or three possible off-target sites with the highest score. The off-target sites were PCR amplified from genomic DNA of gRNA/Cas9-injected embryos and analyzed by direct sequencing. Specific primers for each off-target site were listed on Table S6.

## Additional Information

**How to cite this article**: Hara, S. *et al.* Generation of mutant mice via the CRISPR/Cas9 system using FokI-dCas9. *Sci. Rep.* **5**, 11221; doi: 10.1038/srep11221 (2015).

## Supplementary Material

Supplementary Information

## Figures and Tables

**Table 1:** Production of mice mutated on the *Bcr* locus using fCas9.

**Cas9**	**gRNA**	**Trial**	**Injected/transferred**	**No. of pre- and postnatal embryos (survival rate %^a^)**	**Mutant (%)**	**Monoallelic mutant (%)**	**Biallelic mutant (%)**
fCas9	B1+B2	1	114/103	16 (14.0)	10 (62.5)	8 (80.0)	2 (20.0)
		2	32/26	12 (37.5)	10 (83.3)	9 (92.7)	1 (8.3)
		3	67/58	19 (28.4)	9 (47.3)	9 (100)	0 (0)
		total	213/187	47 (22.1)	29 (61.7)	26 (93.6)	3 (6.4)
D10A	B1+B2	1	54/49	15 (27.8)	5 (33.3)	5 (100)	0 (0)
WT	B1	1	46/34	15 (32.6)	10 (66.7)	10 (100)	0 (0)
	B2	1	40/36	16 (40.0)	10 (62.5)	9 (93.7)	1 (6.3)

Numbers in parentheses represent the percentages calculated from the number of mutants relative to the number of embryos. FokI-dCas9 (fCas9), Cas9 D10A (D10A), Cas9 WT (WT).

^a^Survival rates were calculated from number of embryos relative to number of injected zygotes.

**Table 2:** Production of mice mutated on the *Abl* locus using fCas9.

**Cas9**	**gRNA**	**Trial**	**Injected/ transferred**	**No. of pre- and postnatal embryos (survival rate %^a^)**	**Mutant (%)**	**Monoallelic mutant (%)**	**Biallelic mutant (%)**
fCas9	A1+A2	1	71/54	18 (25.4)	0 (0)	0	0 (0)
		2	61/38	34 (55.7)	0 (0)	0	0 (0)
		total	132/92	52 (39.4)	0 (0)	0	0 (0)
D10A	A1+A2	1	64/54	20 (31.3)	10 (50.0)	10 (100)	0 (0)
WT	A1	1	31/31	10 (32.3)	8 (80.0)	8 (100)	0 (0)
	A2	1	31/30	13 (43.3)	6 (46.2)	5 (92.3)	1 (7.7)

Numbers in parentheses represent the percentages calculated from the number of mutants relative to the number of embryos. FokI-dCas9 (fCas9), Cas9 D10A (D10A), Cas9 WT (WT).

^a^Survival rates were calculated from number of embryos relative to number of injected zygotes.
