# Supplementary material for: Generation of mutant mice via the CRISPR/Cas9 system using FokI-dCas9
Source: Sci Rep. 2015 Jun 9;5:11221. doi: 10.1038/srep11221 (PMC4460908; doi:10.1038/srep11221)
Supplement: Supplementary Information [file srep11221-s1.pdf]

Supplementary Information

Generation of mutant mice via the CRISPR/Cas9 system using FokI-dCas9

Satoshi Hara[1,4], Moe Tamano[1,4], Satoshi Yamashita[1,3], Tomoko Kato[1], Takeshi Saito[1], Tetsushi Sakuma[2], Takashi Yamamoto[2], Masafumi Inui[1] and Shuji Takada[1,*]

[1]Department of Systems BioMedicine, National Research Institute for Child Health and Development, Tokyo 157-8535, Japan; [2]Department of Mathematical and Life Sciences, Graduate School of Science, Hiroshima University, Hiroshima 739-8526, Japan

[3]Present address: Department of Systems BioMedicine, Graduate School of Medical and Dental Sciences, Tokyo Medical and Dental University, Tokyo 113-8510, Japan

[4]These authors contributed equally to this work.

*Correspondence should be addressed to Shuji Takada (takada-s@ncchd.go.jp, 2-10-1 Okura, Setagaya, Tokyo 157-8535, Japan, Tel & Fax: +81-3-3417-2498).

Supplementary Table S1. Comparison of mutation efficiencies using gRNA pairs with different spacer length

Locus	spacer length (bp)	gRNA	Injected / transferred	No. of embryos (survival rate %[a])	Mutant (%)	Monoallelic mutant (%)	Biallelic mutant (%)
Bcr	11	B3+B4	90 / 72	31 (34.4)	0 (0)	0 (0)	0 (0)
	14	B5+B6	44 / 42	8 (18.2)	4 (50.0)	3 (75.0)	1 (25.0)
	16	B1+B2	213 / 187	47 (22.1)	29 (61.7)	26 (93.6)	3 (6.4)
	17	B7+B8	46 / 46	5 (10.9)	0 (0)	0 (0)	0 (0)
	18	B9+B10	45 / 40	9 (20.0)	7 (77.8)	3 (42.9)	4 (57.1)
	19	B11+B12	40 / 36	11 (27.5)	5 (45.5)	3 (60.0)	2 (40.0)
Abl	13	A1+A2	132 / 92	52 (39.4)	0 (0)	0 (0)	0 (0)
	14	A5+A6	45 / 44	10 (22.2)	0 (0)	0 (0)	0 (0)
	16	A3+A4	154 / 72	25 (16.2)	9 (36.0)	9 (100.0)	0 (0)
	17	A7+A8	47 / 44	13 (27.6)	9 (69.2)	5 (55.6)	4 (44.4)
	18	A7+A9	39 / 37	10 (25.6)	7 (70.0)	2 (28.6)	5 (71.4)
	19	A1+A10	43 / 38	7 (16.2)	2 (28.6)	2 (100.0)	0 (0)

Numbers in parentheses represent the percentages calculated from the number of mutants relative to the number of embryos.

[a]Survival rates were calculated from number of embryos relative to number of injected zygotes.

Supplementary Table S2. Mutation efficiencies of B3, B4 and A3 injected with Cas9 WT.

Locus	gRNA	Injected/ transferred	No. of embryos (Survival rate %[a])	Mutant (%)	Monoallelic mutant (%)	Biallelic mutant (%)
Bcr	B3	55 / 36	12 (21.8)	7 (58.8)	7 (100)	0 (0)
	B4	50 / 36	20 (40.0)	2 (10.0)	2 (100)	0 (0)
Abl	A3	49 / 36	15 (30.6)	11 (64.7)	11 (100)	0 (0)

Numbers in parentheses represent the percentages calculated from the number of mutants relative to the number of embryos or newborns.

[a]Survival rates were calculated from number of embryos relative to injected zygotes.

Supplementary Table S3. Off-target analysis of B1 and B2.

target site	Sequence	Chr.	Position (mm9)	Strand	mutation rate (%)				
					gRNA	B1+B2	B1+B2	B1	B2
					Cas9	fCas9	D10A	WT	WT
Bcr_B1	CTATCCATCATCAGATCATTAGG	10	74561466–74561488	-		29/47 (61.7)	5/15 (33.3)	10/15 (66.7)	
Bcr_B1_OT1	CAAGCCAACATCAGATCATTCAG	1	84239159–84239181	+		0/47 (0)	0/15 (0)	0/15 (0)	
Bcr_B1_OT2	CAATACATCGTCAGATCATTCAG	3	112969795–112969817	-		0/47 (0)	0/15 (0)	0/15 (0)	N/A
Bcr_B1_OT3	CTATCCATCATGAGATCATATGG	4	13191165–13191187	-		0/47 (0)	0/15 (0)	0/15 (0)	
Bcr_B2	GCTGGCAGGGCAGTAAAAACAGG	10	74561505–74561527	+		29/47 (61.7)	5/15 (33.3)		10/16 (62.5)
Bcr_B2_OT1	GAAGGCAAGGCAGTAAAAACGAG	X	101766855–101766877	-		0/47 (0)	0/15 (0)	N/A	0/16 (0)
Bcr_B2_OT2	ACTGGCAAGGCAGTAAAAAGAGG	X	83169655–83169677	-		0/47 (0)	0/15 (0)		0/16 (0)
Bcr_B2_OT3	GCAGGCATTGCAGTAAAAACCAG	13	98653829–98653851	-		0/47 (0)	0/15 (0)		0/16 (0)

Numbers in parentheses represent the percentages calculated from the number of mutants relative to the number of embryos.

fCas9 (FokI-dCas9), D10A (Cas9 D10A), WT (Cas9 WT).

Supplementary Table S4. Off-target analysis of A3 and A4.

target site	Sequence	Chr.	Position (mm9)	Strand	mutation rate (%)		
					gRNA	A3+A4	A3
					Cas9	fCas9	WT
Abl_A3	ACTACTCAGCAGTATGTTCTAGG	2	31621244–31621266	-		9/25 (36.0)	11/15 (73.3)
Abl_A3_OT1	ACTACTGAGCAGCATGTTCTGAG	8	117086071–117086093	-		0/25 (0)	0/15 (0)
Abl_A3_OT2	ACTCCTCCTCAGTATGTTCTGAG	6	49304224–49304246	+		0/25 (0)	0/15 (0)
Abl_A4	GTGGGCAGGAAAGGAAGAGGAGG	2	31621283–31621305	+		9/25 (36.0)	N/A
Abl_A4_OT1	GTGGGCAGGACAGGAAGAGGGGG	5	33376892–33376914	-		0/25 (0)	
Abl_A4_OT2	GGGGGCAGGAAAGGAACAGGAGG	16	4912768–4912790	-		0/25 (0)	
Abl_A4_OT3	GAGGGCGGGAAAGGAAGAGGAGG	9	108392953–108392975	-		0/25 (0)	

Numbers in parentheses represent the percentages calculated from the number of mutants relative to the number of embryos.

fCas9 (FokI-dCas9), WT (Cas9 WT).

Supplementary Table S5. Comparison of mutation efficiencies using TALENs.

Locus	TALEN	Injected/ transferred	No. of embryos (survival rate %)	Mutant (%)	Monoallelic mutant (%)	Biallelic mutant (%)
Bcr	Platinum	78/63	26 (33.3)	13 (50.0)	13 (100)	0 (0)
	Golden	94/80	13 (13.8)	3 (23.0)	3 (100)	0 (0)
Abl	Platinum	80/52	17 (21.3)	11 (64.7)	11 (100)	0 (0)
	Golden	90/77	45 (50.0)	0 (0)	0 (0)	0 (0)

Numbers in parentheses represent the percentages calculated from the number of mutants relative to the number of embryos.

[a]Survival rates were calculated from number of embryos relative to injected zygotes.
Platinum Gate TALEN (Platinum), Golden Gate TALEN (Golden).

Supplementary Table S6. Primer sequences

Procedure	Primer set	Forward (5' -3')	Reverse (5'-3')
gRNA cloning	B1	TATCCATCATCAGATCATTGTTTTAGAGCTAGAAATAGCAAG	AACAATGATCTGATGATGGATACGGTGTTTCGTCCTTTCCAC
	B2	CTGGCAGGGCAGTAAAAACGTTTTAGAGCTAGAAATAGCAAG	AACGTTTTTACTGCCCTGCCAGCGGTGTTTCGTCCTTTCCAC
	B3	ATCAGATCATTAGGACCACGTTTTAGAGCTAGAAATAGCAAG	AACGTGGTCCTAATGATCTGATCGGTGTTTCGTCCTTTCCAC
	B4	ACCCAGGGTCTGGCTGGCAGTTTTAGAGCTAGAAATAGCAAG	AACTGCCAGCCAGACCCTGGGTCGGTGTTTCGTCCTTTCCAC
	B5	GACCACAGGCATGACAGAAGTTTTAGAGCTAGAAATAGCAAG	AACTTCTGTCATGCCTGTGGTCCGGTGTTTCGTCCTTTCCAC
	B6	GATGGACCCAGGGTCTGGCGTTTTAGAGCTAGAAATAGCAAG	AACGCCAGACCCTGGGTCCATCCGGTGTTTCGTCCTTTCCAC
	B7	CAGGCATGACAGAACGGGCGTTTTAGAGCTAGAAATAGCAAG	AACGCCCGTTCTGTCATGCCTGCGGTGTTTCGTCCTTTCCAC
	B8	ATGATGGATAGATGGACCCGTTTTAGAGCTAGAAATAGCAAG	AACGGGTCCATCTATCCATCATCGGTGTTTCGTCCTTTCCAC
	B9	GCCCTGCCAGCCAGACCCTGTTTTAGAGCTAGAAATAGCAAG	AACAGGGTCTGGCTGGCAGGGCCGGTGTTTCGTCCTTTCCAC
	B10	ATCTTTTGAAAATAATCCAGTTTTAGAGCTAGAAATAGCAAG	AACTGGATTATTTTCAAAAGATCGGTGTTTCGTCCTTTCCAC
	B11	GAACGGGCTGGCACGCTGCGTTTTAGAGCTAGAAATAGCAAG	AACGCAGCGTGCCAGCCCGTTCCGGTGTTTCGTCCTTTCCAC
	B12	TGATCTGATGATGGATAGAGTTTTAGAGCTAGAAATAGCAAG	AACTCTATCCATCATCAGATCACGGTGTTTCGTCCTTTCCAC
	A1	AGCAGTATGTTCTAGGAAGGTTTTAGAGCTAGAAATAGCAAG	AACCTTCCTAGAACATACTGCTCGGTGTTTCGTCCTTTCCAC
	A2	AGTGGAGAGTGGGCAGGAAGTTTTAGAGCTAGAAATAGCAAG	AACTTCCTGCCCACTCTCCACTCGGTGTTTCGTCCTTTCCAC
	A3	CTACTCAGCAGTATGTTCTGTTTTAGAGCTAGAAATAGCAAG	AACAGAACATACTGCTGAGTAGCGGTGTTTCGTCCTTTCCAC
	A4	TGGGCAGGAAAGGAAGAGGGTTTTAGAGCTAGAAATAGCAAG	AACCCTCTTCCTTTCCTGCCCACGGTGTTTCGTCCTTTCCAC
	A5	GAGTGGGCAGGAAAGGAAGGTTTTAGAGCTAGAAATAGCAAG	AACCTTCCTTTCCTGCCCACTCCGGTGTTTCGTCCTTTCCAC
	A6	CAAAAGTAAAGGGCTGGCTGTTTTAGAGCTAGAAATAGCAAG	AACAGCCAGCCCTTTACTTTTGCGGTGTTTCGTCCTTTCCAC
	A7	CACAATGAGAACTGTTACTGTTTTAGAGCTAGAAATAGCAAG	AACAGTAACAGTTCTCATTGTGCGGTGTTTCGTCCTTTCCAC
	A8	TTTCCCAGAGGAAAGAAATGTTTTAGAGCTAGAAATAGCAAG	AACATTTCTTTCCTCTGGGAAACGGTGTTTCGTCCTTTCCAC
	A9	GGTGTGCCGACTCTCCTCAGTTTTAGAGCTAGAAATAGCAAG	AACTGAGGAGAGTCGGCACACCCGGTGTTTCGTCCTTTCCAC
	A10	GTGTGCCGACTCTCCTCATGTTTTAGAGCTAGAAATAGCAAG	AACATGAGGAGAGTCGGCACACCGGTGTTTCGTCCTTTCCAC
Template for gRNA synthesis	T7_B1	TTAATACGACTCACTATAGGTATCCATCATCAGATCATT	AAAAGCACCGACTCGGTGCC
	T7_B2	TTAATACGACTCACTATAGGCTGGCAGGGCAGTAAAAAC	
	T7_B3	TTAATACGACTCACTATAGGATCAGATCATTAGGACCAC	
	T7_B4	TTAATACGACTCACTATAGGACCCAGGGTCTGGCTGGCA	
	T7_B5	TTAATACGACTCACTATAGGGACCACAGGCATGACAGAA	
	T7_B6	TTAATACGACTCACTATAGGGATGGACCCAGGGTCTGGC	
	T7_B7	TTAATACGACTCACTATAGGCAGGCATGACAGAACGGGC	
	T7_B8	TTAATACGACTCACTATAGGATGATGGATAGATGGACCC	
	T7_B9	TTAATACGACTCACTATAGGGCCCTGCCAGCCAGACCCT	
	T7_B10	TTAATACGACTCACTATAGGATCTTTTGAAAATAATCCA	
	T7_B11	TTAATACGACTCACTATAGGGAACGGGCTGGCACGCTGC	
	T7_B12	TTAATACGACTCACTATAGGTGATCTGATGATGGATAGA	
	T7_A1	TTAATACGACTCACTATAGGAGCAGTATGTTCTAGGAAG	
	T7_A2	TTAATACGACTCACTATAGGAGTGGAGAGTGGGCAGGAA	
	T7_A3	TTAATACGACTCACTATAGGCTACTCAGCAGTATGTTCT	
	T7_A4	TTAATACGACTCACTATAGGTGGGCAGGAAAGGAAGAGG	
	T7_A5	TTAATACGACTCACTATAGGGAGTGGGCAGGAAAGGAAG	
	T7_A6	TTAATACGACTCACTATAGGCAAAAGTAAAGGGCTGGCT	
	T7_A7	TTAATACGACTCACTATAGGCACAATGAGAACTGTTACT	
	T7_A8	TTAATACGACTCACTATAGGTTTCCCAGAGGAAAGAAAT	
	T7_A9	TTAATACGACTCACTATAGGGGTGTGCCGACTCTCCTCA	
	T7_A10	TTAATACGACTCACTATAGGGTGTGCCGACTCTCCTCAT	
Template for Cas9 mRNA synthesis	T7-Cas9	TAATACGACTCACTATAGGGAGAATGGACAAGAAGTACTCCATTGG	TCACACCTTCCTCTTCTTC
Genotyping	Bcr	CAGGGAGGCAGGAGTGTTTAC	TCAAAAGATTCGGTTTCCTG
	Abl	CAGCCAGCCCTTTACTTTTG	ATGCATACACACAGGCAGGA
Off-target analysis	B1_OT1	GCACTCAGGTCCCCACTAGA	TGCAACCAGAAGCAAGTTGT
	B1_OT2	CCCATTTCTGTTCCTCTCCA	CCAGGCTGGATCACATAACA
	B1_OT3	TGGCAGGTAATGGCTAGGTC	TCACCAGTTCCAAGGAGAGG
	B2_OT1	TCTTCCTTCAGTTCTTAGCTTATTCC	AGAGTTCAAGAACAGCCAAGG
	B2_OT2	TGGCAAGGTGAGAGATAATCAG	AGGCTTCGGTAACACAGAGG
	B2_OT3	CAGAAACCCAAGGGACCATA	TGTTCCAGCAGAGTCGAATG
	A3_OT1	TAAGCCCATCCAGTCCAAGT	GCACCGTCTTGTCTCCTAGC
	A3_OT2	CCTCTTCAGCCTCCCTCTCT	CAGGCCCACAACACTCTTTT
	A4_OT1	CGCCATCCAGTCCTGTAACT	CGACACCACTTGTCATCCAC
	A4_OT2	ACCCTGACCCATGCTTACTG	GCCACCAGATGGGCTAGATA
	A4_OT3	GGCAACACGCTCTCGATT	CATCAACTTGCAGGTCCAGA
